# Case Report: Endoscopic cystectomy vs. lumbar interbody fusion for liquid- and gas-filled discal cysts: a case series and literature review

**DOI:** 10.3389/fsurg.2025.1646605

**Published:** 2025-09-11

**Authors:** Haoyun Huang, Guangye Li, Junwen Deng, Rigao Chen, Yi Zhou

**Affiliations:** ^1^ School of Clinical Medicine, Chengdu University of Traditional Chinese Medicine, Chengdu, China; ^2^ Department of Orthopedics, Hospital of Chengdu University of Traditional Chinese Medicine, Chengdu, China

**Keywords:** discal cyst, gas-filled, liquid-filled, endoscopic cystectomy, lumbar interbody fusion, case report

## Abstract

Lumbar discal cysts are uncommon lesions that mimic disc herniation but require distinct therapeutic strategies because of their unique pathophysiologies. However, the current literature lacks consensus on the adoption of optimal surgical approaches. This study reviews the surgical management and pathological mechanisms of primary lumbar discal cysts, emphasizing the distinction between liquid- and gas-filled subtypes that require tailored therapeutic strategies due to their differing pathophysiologies and association with spinal instability. We report successful surgical outcomes in three patients: one with a liquid cyst treated with endoscopic resection and two with gas-filled cysts managed with endoscopic cystectomy or lumbar interbody fusion, respectively. All patients experienced significant symptomatic relief and complete cyst resolution on imaging. A concurrent PubMed literature review (1990–2025) on primary gas-filled and liquid disc cysts informed the analysis. Liquid cysts predominantly occur in young patients, and these are associated with annular fiber damage and disc herniation, causing symptoms primarily through direct compression; endoscopic cystectomy is an effective treatment. Conversely, gas-filled cysts are more common in old patients, and these are strongly linked to disc degeneration and the vacuum phenomenon. Symptoms arise not only from cyst compression but also potentially from concurrent spinal stenosis and vertebral instability. Therefore, the surgical strategy for gas-filled cysts must consider the factor of spinal stability: endoscopic cystectomy is suitable for stable spines, while interbody fusion surgery is more appropriate when instability is present. We conclude that surgical intervention is effective for disc cysts, but the optimal approach must be individualized on the basis of cyst subtype and the presence of spinal instability, as informed by clinical presentation and imaging features.

## 1 Introduction

Lumbar disc cysts are a rare cause of lumbar radiculopathy, exhibiting a significant gender and geographic predilection, with a higher prevalence reported among Asian males (1). The pathogenesis is closely linked to intervertebral disc annulus fibrosus injury and degenerative changes (2). Typical clinical manifestations include radiating pain and sensory abnormalities within the affected dermatome of the nerve root, often accompanied by signs of nerve compression such as neurogenic intermittent claudication (3, 4). The clinical presentation of lumbar disc cysts is challenging to distinguish from other causes of lumbar canal stenosis, including disc herniation, tumors, synovial cysts, and hematomas. This overlap in clinical and radiological features can complicate timely diagnosis and management (5). Clinically, these cysts are categorized into two subtypes on the basis of cyst content: liquid-filled and gas-filled. Surgery is an established and effective treatment for symptomatic disc cysts. However, due to the rarity of the condition, its pathogenesis remains incompletely elucidated, and the factors distinguishing the subtypes continue to be debated (6). Consequently, consensus regarding optimal surgical approaches is lacking in the existing literature.

Therefore, this study reports the surgical management and outcomes of three patients with lumbar disc cysts: one liquid-filled cyst treated with endoscopic cystectomy and two gas-filled cysts managed with endoscopic cystectomy and lumbar interbody fusion, respectively. All patients experienced significant symptomatic relief postoperatively, with imaging follow-up confirming complete cyst resolution. Furthermore, by reviewing pertinent literature, we aimed to analyze and summarize the distinct pathophysiological characteristics of the different cyst subtypes and explore the most appropriate surgical interventions accordingly.

## 2. Case presentation

### 2.1 Case 1

**Figure 1:**
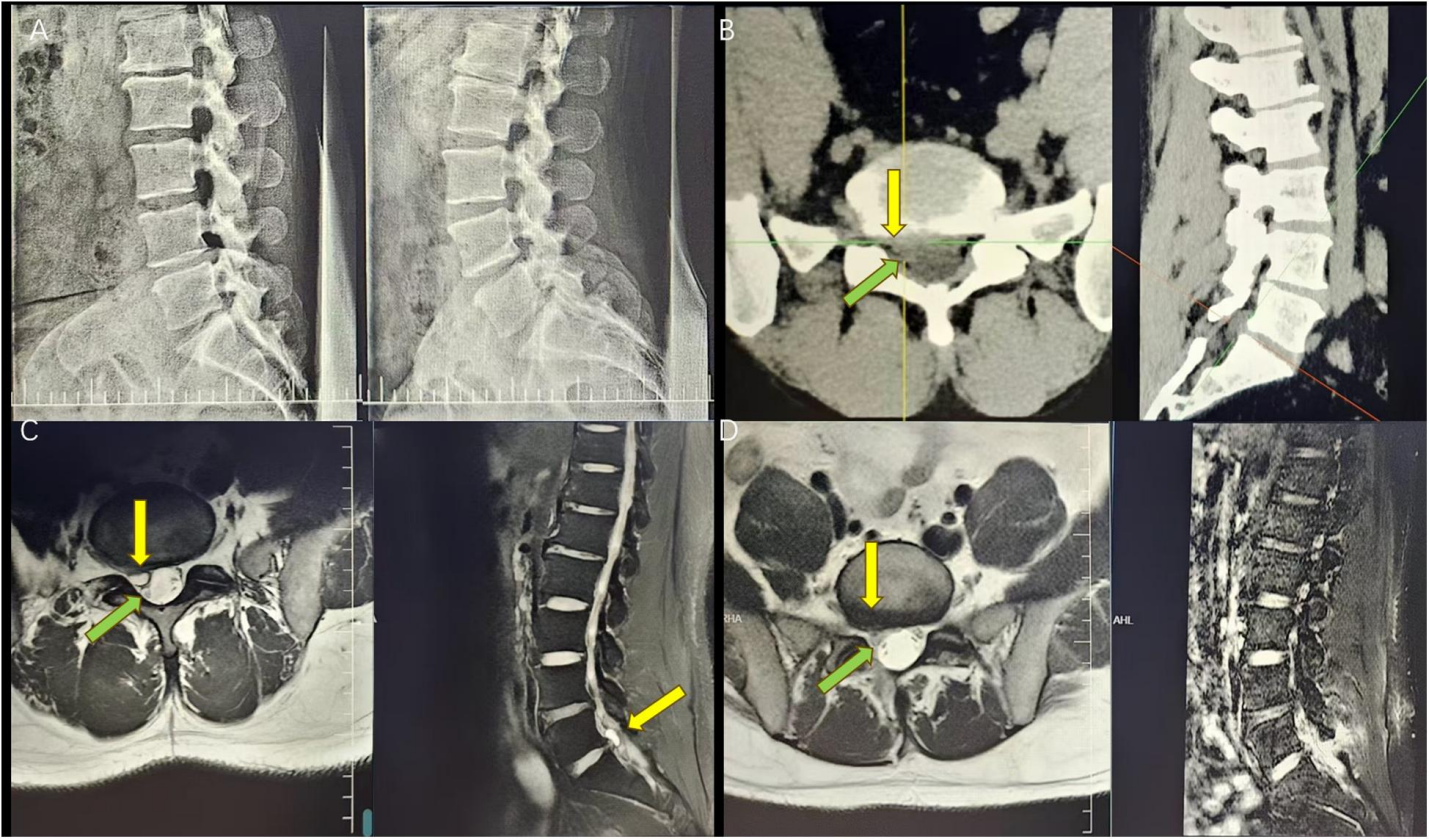
**(A)** Preoperative digital radiography (DR) showing normal intervertebral height and vertebral stability. **(B)** Preoperative CT showing a herniated disc on the right side of L5/S1 with the corresponding nerve root compression (the yellow arrow indicates the location of the herniated disc and the green arrow indicates the location of the compressed nerve root emanation). **(C)** Preoperative MRI showing a dense T1/T2 image with the corresponding nerve root compression (the yellow arrow indicates the location of the herniated disc and the green arrow indicates the location of the compressed nerve root emanation). **(D)** Postoperative MRI suggesting disappearance of the cyst and nerve root relaxation (the yellow arrow indicates the location of the original cyst and the green arrow shows nerve root relaxation).

**Figure 2:**
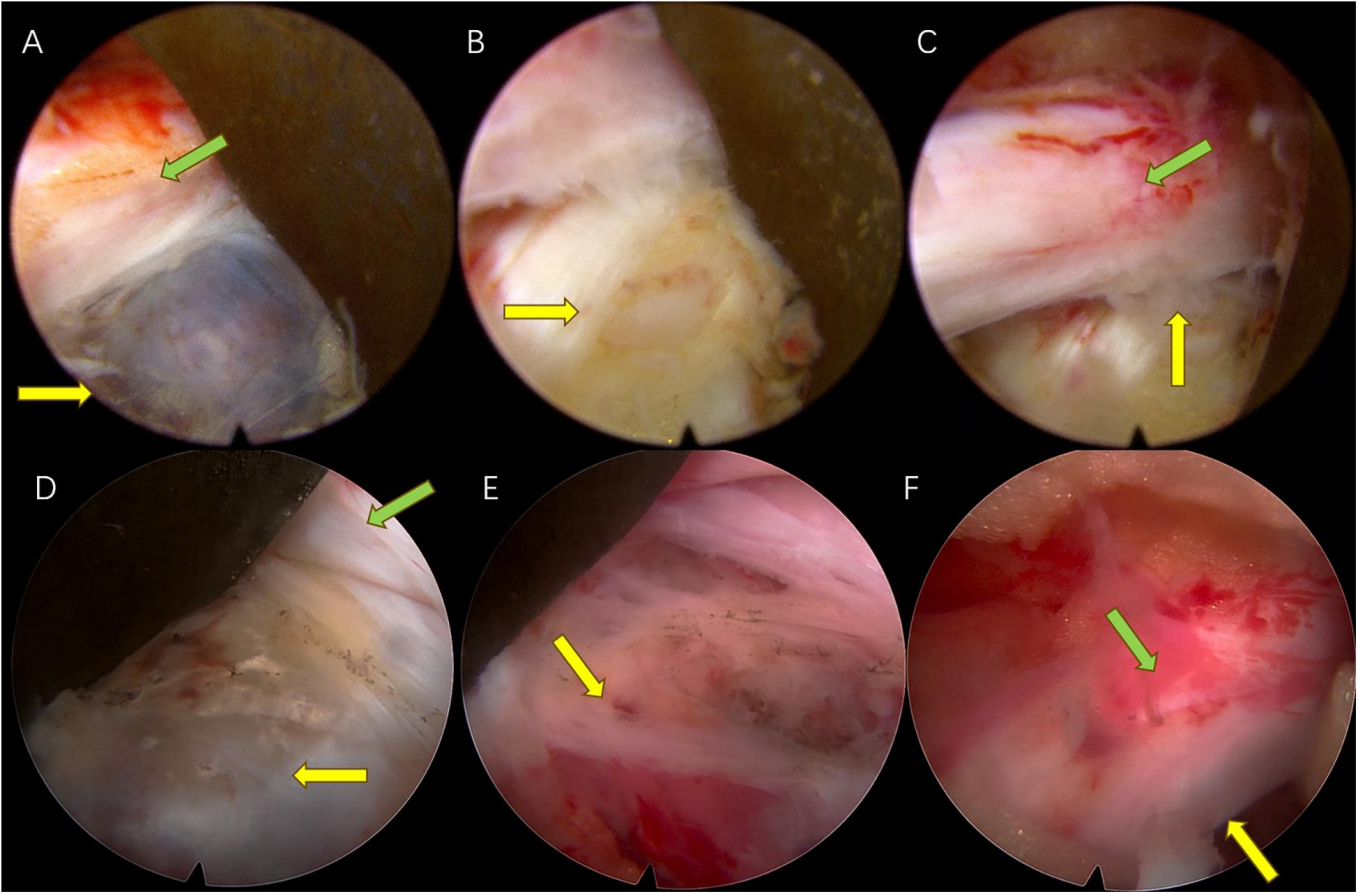
Case 1: **(A)** Endoscopic view of the cyst compressing the S1 nerve root (the yellow arrow indicates the location of the cyst and the green arrow indicates the compressed nerve root). **(B)** A breach in the fibrous ring connecting to the cyst is seen after cyst resection (the yellow arrow indicates the location of the original cyst). **(C)** The nerve is completely released after removal of the cyst and the protruding nucleus pulposus (the yellow arrow indicates the original location of the cyst and the green arrow shows nerve root relaxation). Case 2: **(D)** Endoscopic view of the cyst compressing the S1 nerve root (the yellow arrow indicates the location of the cyst and the green arrow indicates the compressed nerve root). **(E)** A breach in the fibrous ring connecting to the cyst is seen after cyst removal (the yellow arrow indicates the location of the original cyst). **(F)** The nerve is completely relieved after removal of the cyst and the protruding nucleus pulposus (the yellow arrow indicates the original location of the cyst and the green arrow shows nerve root relaxation).

The patient was a 41-year-old man who presented with radiating pain and sensory abnormality in the right lower limb for over 1 month, with a progressive aggravation of symptoms [visual analog scale (VAS) score = 7]. The patient denied any history of previous lumbar trauma or surgery. A neurological examination showed a positive right Lasegue's test (45°). A lumbar spine dynamic x-ray showed a normal image with no signs of lumbar instability, deformity, or loss of intervertebral height (Figure 1A). Lumbar spine computed tomography suggested a right-sided herniation of the L5/S1 intervertebral disc with an obvious compression of the S1 nerve root (Figure 1B). Magnetic resonance imaging (MRI) suggested that the right posterior margin of the L5/S1 intervertebral disc had low-density shadows in the T1 sequence and high-density shadows in the T2 sequence, and fluid cystic lesions were present, with a compression of the adjacent dural sac and the right S1 nerve root (Figure 1C). The patient underwent endoscopic cystectomy with an intraoperative release of bloody, yellowish fluid and complete removal of the cystic tissue (Figures 2A–C). The patient experienced immediate postoperative symptomatic relief (VAS score = 1), and a postoperative MRI showed complete disappearance of the cyst (Figure 1D). A pathological biopsy showed fibrous and vascular tissue in the cyst wall, fibrous tissue hyperplasia with hyaline lesions, and focal old hemorrhage (Supplementary Figure S1). At 1-, 3-, and 6-month follow-ups, the patient reported that the original symptoms and signs completely disappeared (VAS score = 0).

### 2.2 Case 2

**Figure 3:**
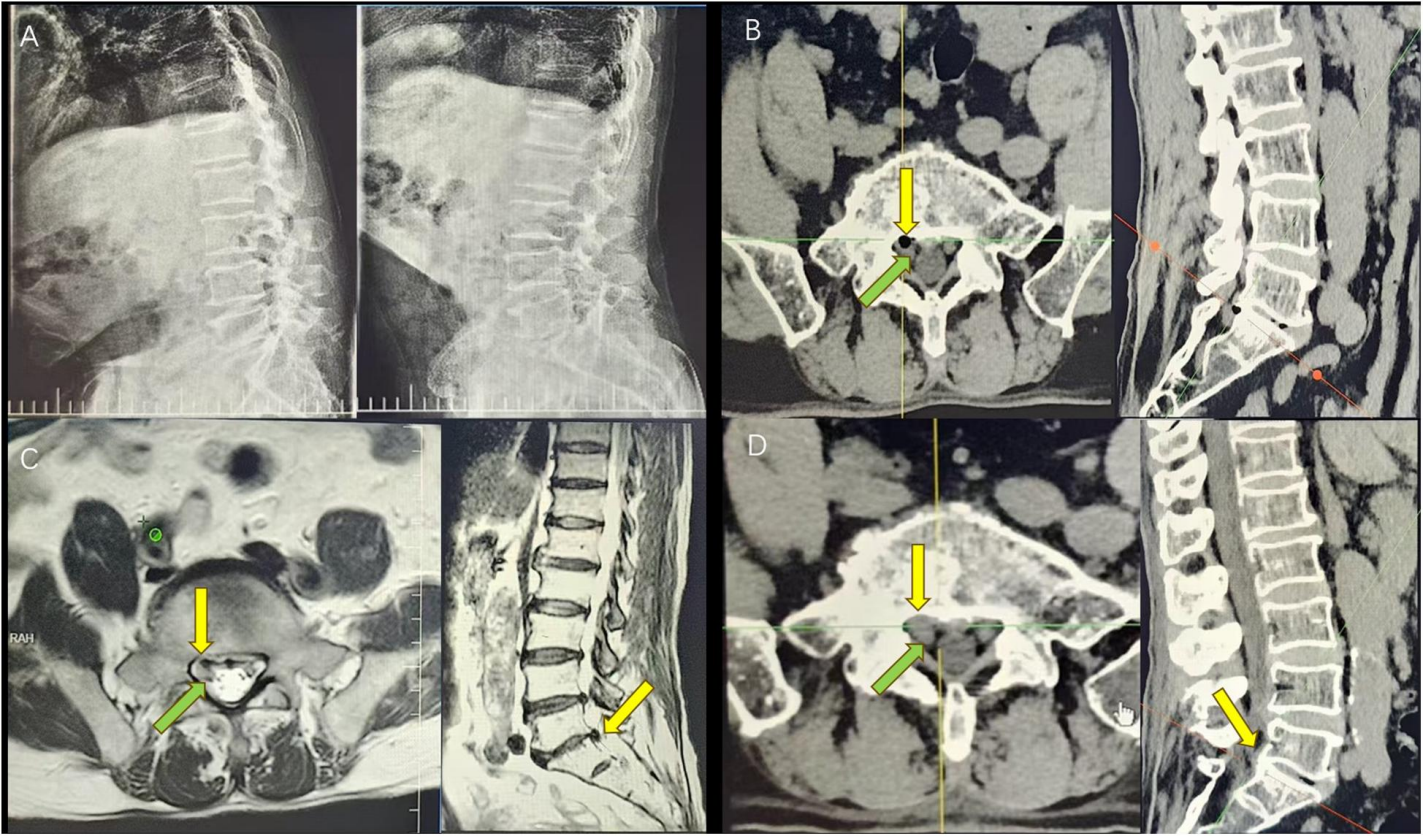
**(A)** Preoperative DR shows that the height of the L5/S1 intervertebral space is reduced and the vertebral body is still stable. **(B)** Preoperative CT shows an L5/S1 disc vacuum sign, right disc herniation with vacuoles (the yellow arrow indicates the location of the herniated disc), and a compression of the corresponding nerve root (the green arrow indicates the location of the compressed nerve root emanation). **(C)** Preoperative MRI shows a T1/T2 low hyperdensity shadow (the yellow arrow indicates the location of the herniated disc) and the corresponding compressed nerve root (the green arrow indicates the location of the compressed nerve root emanation). **(D)** Postoperative CT suggesting disappearance of the cyst (the yellow arrow suggests the location of the original cyst) and relaxation of the nerve root (the green arrow shows nerve root relaxation).

This patient was a 68-year-old man with numbness and pain in the right lower limb for 1 year, aggravated for 20 days (VAS score = 6). The patient denied any history of previous lumbar trauma or surgery. A neurological examination revealed a positive right Lasegue's test (60°). A lumbar spine dynamic x-ray suggested reduced intervertebral height at L5/S1 and acceptable lumbar spine stability (Figure 3A). CT suggested disc herniation and a low-density air bubble shadow in the right lower part of the L5/S1 disc, an obvious compression of the S1 nerve root, a disc vacuum phenomenon, and an obvious reduction of intervertebral height (Figure 3B). MRI suggested hypodense T1 and T2 sequential shadows on the right side of the L5/S1 disc (Figure 3C), and a gas-filled cyst was present. The patient was treated with endoscopic cystectomy, during which a large number of air bubbles and a small amount of bloody, yellowish fluid were released and the cystic tissue was removed by preserving its intactness (Figures 2D,E). The patient's symptoms resolved immediately after surgery (VAS score = 2), and a postoperative CT suggested complete disappearance of the cyst (Figure 3D). At 1-, 3-, and 6-month follow-ups, the patient's original symptoms and signs completely disappeared (VAS score = 0).

### 2.3 Case 3

**Figure 4:**
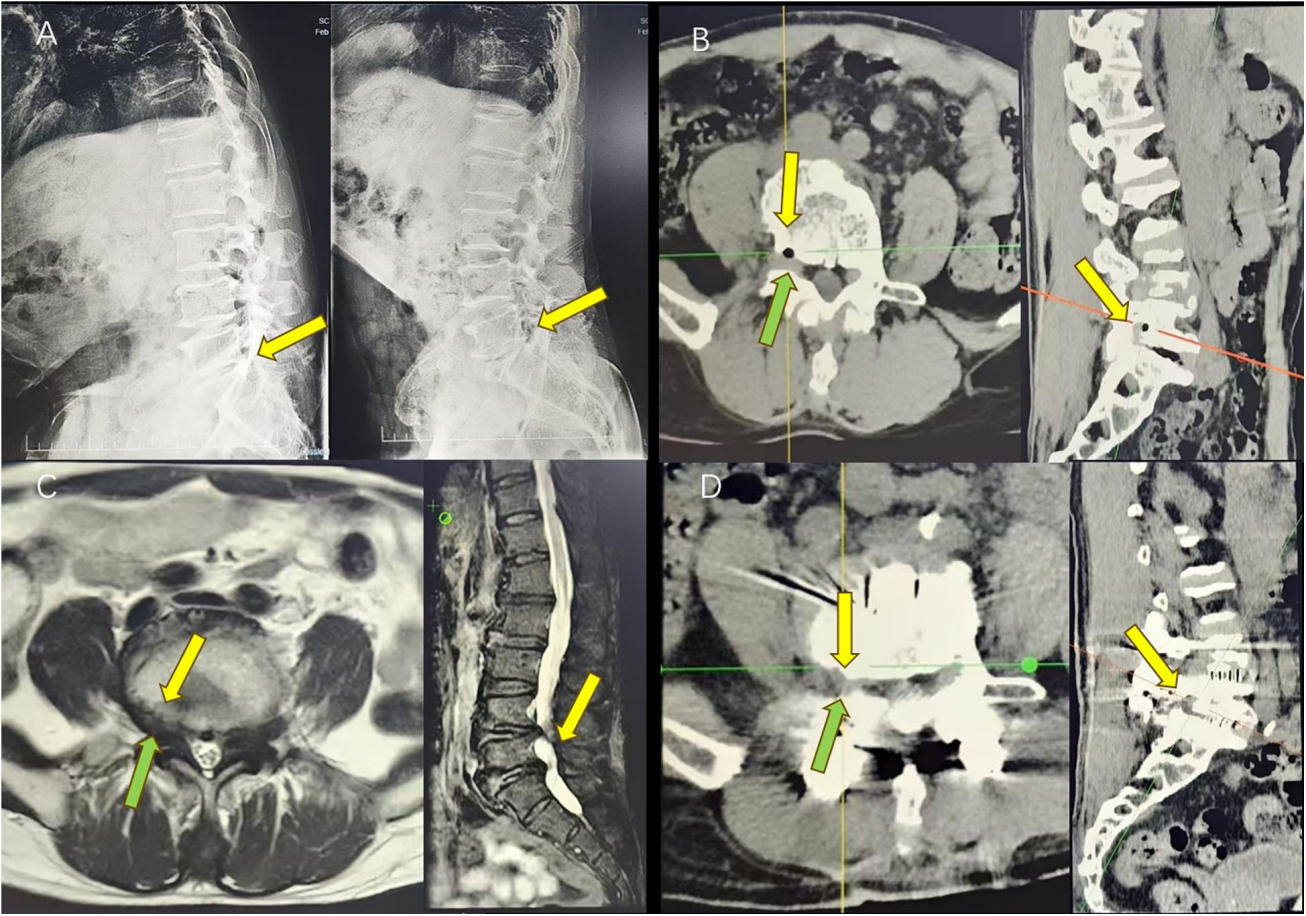
**(A)** Preoperative DR shows a reduction of the intervertebral heights of L4/5 and L5/S1 and I° slip of the L5/S1 vertebral body (yellow arrow). **(B)** Preoperative CT suggests that the L5/S1 disc is vacuumed, and the right disc is herniated with a vacuole (the yellow arrow indicates the location of the herniated disc) and the corresponding nerve root is compressed (the green arrow indicates the location of the compressed nerve root emanation). **(C)** Preoperative MRI shows T1/T2 hypodensities (the yellow arrow indicates the location of the herniated disc), with the corresponding nerve root compression (the green arrow indicates the location of the compressed nerve root emanation). **(D)** Postoperative CT shows the disappearance of the cyst (the yellow arrow indicates the location of the original cyst), with nerve root relaxation (the green arrow shows nerve root relaxation).

This patient was a 74-year-old man, with low back pain for 4 years, aggravated with numbness and pain in the right lower limb for 6 months, worsened by position-related symptoms, and neurogenic intermittent claudication. The symptoms include increased pain, numbness, or weakness in the lower limbs, which can hinder ambulation and can be mitigated by rest for about 200 m (VAS score = 7). The patient denied any history of previous lumbar trauma or surgery. A neurological examination showed a positive right Lasegue's test (60°). A lumbar spine dynamic x-ray suggested a significant decrease in the intervertebral height of L4/5 and L5/S1, lumbar instability, and a posterior I° slip of the L5 vertebral body (Figure 4A). CT suggested a right-sided hypodense air bubble shadow of the L5/S1 disc, L5 nerve root compression, and L4/5 and L5/S1 disc vacuum signs (Figure 4B). MRI suggested hypodense T1 and T2 sequential shadows on the right side of the L5/S1 disc (Figure 4C). The patient was treated with L4/5 and L5/S1 interbody fusion, during which the degenerative disc and cyst tissue were completely removed. The patient's symptoms were relieved immediately after surgery (VAS score = 2), and a repeat CT suggested that the cyst had disappeared, and the intervertebral height and vertebral stability were restored (Figure 4D). At the 1-month follow-up, the patient's original symptoms and signs relieved (VAS score = 1). At the 3- and 6-month follow-ups, the patient's lower limb symptoms were completely relieved (VAS score = 0). Table 1 provides the basic characteristics of the patient cases.

**Table 1:** Basic characteristics of the patients in our series.

Case no.	Age/sex	Level	Symptoms	Type	Vacuum sign of the intervertebral disc	MRI T1	MRI T2	Surgical strategy
1	41/Male	L5/S1	Radiating pain and numbness in the right lower limb	Liquid-filled	No	Low signal	High signal	Endoscopic cystectomy
2	68/Male	L5/S1	Radiating pain and numbness in the right lower limb	Gas-filled	Yes	Low signal	Low signal	Endoscopic cystectomy
3	74/Male	L5/S1	Radiating pain and numbness in the right lower limb, accompanied by neurogenic claudication.	Gas-filled	Yes	Low signal	Low signal	Transforaminal lumbar interbody fusion

## 3 Discussion

### 3.1 Literature review

We searched the PubMed database using keywords such as “discal cyst”, “disc cyst”, “spinal cyst”, “vertebral cyst”, and “epidural air cyst”. The literature related to the etiology, pathology, and surgical treatment of primary disc cysts published from January 1990 to March 2025 was searched and reviewed (Supplementary Table S1) (1, 3, 6–32).

### 3.2 Pathology and pathogenesis

Intervertebral disc cysts are extremely rare lesions with significant subtype differences in their mechanism of occurrence. Fluid disc cysts were first identified by Chiba in 2001 and described as cysts directly associated with the corresponding discs (2). These cysts are most commonly seen in young male patients, and their clinical presentation is very similar to that of disc herniation, with both presenting primarily as radicular pain secondary to nerve root compression (4). It is now believed that the cyst is mostly closely associated with disc herniation. A case reported by Bansil et al. found a patient with a disc herniation that transformed into a disc cyst after 6 months of follow-up (20). Kim found, through pathological studies, that the wall of the cysts contains the peritoneum and the relatively hard outer layer over the inner layer (17). All of this evidence supports the theory that the cyst originated from a herniated disc. However, its exact pathogenesis remains controversial. Chiba et al. proposed the theory of epidural hematoma, suggesting that epidural hematoma formation due to bleeding from the epidural venous plexus as a result of injury is the main cause of cyst formation (2). Toyama suggested that ruptured epidural venous plexus hemorrhage may initially be caused by mechanical irritation from disc herniation (33). This theory elucidates the reason why the contents of fluid cysts are mostly bloody, but it is difficult to explain the phenomenon that mostly pathways are seen between the cysts and the intervertebral discs. Kono et al. have a different view on this, suggesting that disc cysts are caused by focal degeneration of a herniated disc, with the herniated tissue causing localized aseptic inflammation, leading to fluid exudation and pseudomembrane formation, and ultimately to the development of a liquid-filled cyst (34). Msheik et al, on the other hand, pointed out by reviewing previous pathological findings that although disc cysts are closely associated with the disc, the cysts are completely devoid of disc material (7). Based on our intraoperative findings, we discovered that the contents of the cyst were mainly yellowish fluid and bloody fluid, and that the cyst itself was connected to the fibrous annulus rupture. These pathological findings indicated the cystic wall tissue to be characterized by fibrous and vascular tissue, in addition to focal old hemorrhages. However, no disc material was observed. Consequently, we believe that the first mechanism may play a major role in the formation of liquid-filled cysts. In summary, the pathophysiology is as follows: a herniated intervertebral disc causes mechanical irritation, which, in turn, results in the rupture and bleeding of the epidural venous plexus. The incomplete resorption of the hematoma that follows leads to cyst formation.

Pneumatic disc cysts are even rare and are considered a rare complication of the disc vacuum phenomenon (35). The intervertebral disc vacuum phenomenon is closely related to disc degeneration (36), which is common in middle-aged and elderly people. Approximately 50% of patients over 40 years are reported to have varying degrees of disc vacuum disease (37). As the degeneration increases, fissures form within the disc, and the expanding fissure creates negative pressure, attracting the surrounding tissues to release nitrogen air to accumulate in it. As the lumbar spine continues to move, the air that accumulates in the disc may migrate toward the disc margins through the “valve pump” mechanism, and when the fibrous annulus tears but does not rupture completely, the air may herniate into the spinal canal at the weak point and form a gas-filled cyst (38). Based on our findings on preoperative imaging, we discovered that in our study, the condition of the two patients with gas-filled cysts was accompanied by a severe disc vacuum and significant intervertebral height reduction. Intraoperatively, when the gas-filled cyst was punctured, a large number of air bubbles with a small amount of bloody and yellowish fluid were released (Case 2), which was consistent with the mechanism described above, suggesting that gas-filled cysts are closely related to disc degeneration.

### 3.3 Surgical treatment

A review of the previous literature suggests that surgery is a reasonable modality for the treatment of disc cysts; however, the optimal surgical indications and protocols remain to be investigated (39). Wang et al. reviewed nine successful cases of microscopic cystectomy and found that the symptoms of patients with cysts were similar to those with lumbar disc herniation and thus, they concluded that the indications for the surgical treatment of disc cysts were similar to those for lumbar herniation treatment (24,25). Chen et al. reviewed nine clinical cases of endoscopic cystectomy for disc cysts and pointed out that when a disc cyst is surgically removed, the whole cyst should be removed together with the disc rupture at the base of the cyst to reduce the probability of recurrence (15).

Surgical treatment of disc cysts includes microscopic or endoscopic cystectomy. Park et al. reported that microscopic cystectomy is a simple and effective surgical procedure with a low recurrence rate (16). Suo et al. reported two successful cases of endoscopic removal of disc cysts, suggesting that spinal endoscopy is an effective modality for minimally invasive removal of disc cysts (14). Our surgical experience has also shown that endoscopic cystectomy is an effective treatment for disc cysts because it offers clear vision, safety, and soundness.

However, simply performing cystectomy will not be effective for all patients. Wang et al. reported the case of a patient with intervertebral disc cyst with lumbar spondylolisthesis, suggesting that the stress imbalance caused by lumbar instability was a possible cause of the cyst, and a positive result was achieved after performing cystectomy with interbody fusion (40). For patients with fluid cysts, as shown in Case 1, who have the characteristics of young age, better disc quality, and better spinal stability, and whose neurological symptoms are mostly due to a mechanical compression of the cysts, endoscopic cystectomy can be performed to obtain a good outcome. However, for patients with gas-filled disc cysts, which are mostly accompanied by severe disc degeneration, poor disc quality, and spinal instability, the symptoms may be associated with lumbar instability and other abnormalities, in addition to mechanical compression, and therefore, appropriate surgical methods need to be selected according to the actual condition of these patients. In patients with simple cystic compression, cystectomy is effective. In patients with gas-filled cysts with significant lumbar instability, interbody fusion surgery may be a more reasonable option if imaging studies are clearly suggestive of lumbar spondylolisthesis, i.e., dynamic x-rays showing motion >4 mm or angulation >10° (Case 3).

### 3.4 Choice of surgical treatment

Surgical removal is a good treatment for primary disc cysts. However, simple cystectomy is not effective in all patients, and an appropriate surgical plan should be selected on the basis of the nature of the cyst and the source of the patient's symptoms.

In patients with liquid-filled cysts, the etiology is closely related to disc herniation, as they tend to occur in young and middle-aged adults. Radiographic examination suggests a compression of the responsible nerve by the cyst accompanied by the condition of normal stability of the lumbar spine. Symptoms are mostly caused by a mechanical compression of the cyst. Patients with such cysts have relatively good disc quality and normal lumbar spine stability, and endoscopic cystectomy can achieve good results.

In patients with gas-filled cysts, the etiology of the disease is mostly associated with degenerative disc disease and a disc vacuum sign, and it occurs in middle-aged and elderly patients. In addition to a compression of the responsible nerve by the cyst, imaging studies also show disc vacuum signs, and some patients have a combination of lumbar spinal stenosis and lumbar spine instability. Symptoms may be associated with lumbar spinal stenosis and lumbar spinal instability in addition to a mechanical compression of the cyst alone. Such patients tend to have poor disc quality, and endoscopic cystectomy may be an option if symptoms are caused only by cystic compression and if an imaging test suggests normal lumbar spine stability. If the patient's symptoms are accompanied by features of lumbar instability such as neurogenic claudication, and an imaging test suggests lumbar instability or slippage, lumbar interbody fusion surgery may be a better option.

There are some limitations in this study. First, the sample size of this study was small, with all three cysts occurring in the L5/S1 segment in males. This limits the generalizability of the findings. Second, available studies have shown that cystectomy is effective in patients with simple cysts. However, for patients with cysts combined with spondylolisthesis, the optimal surgical plan remains to be further investigated. Further clinical studies with larger sample sizes are required to validate our findings.

## 4 Conclusion

Surgical treatment is effective for disc cysts. However, the specific surgical method and strategy should be personalized on the basis of the type of cyst and patient symptoms. Endoscopic cystectomy is a suitable treatment for liquid- and gas-filled cysts with lumbar stability, but for those with lumbar instability, lumbar interbody fusion may be a better choice. Reviewing the cases elucidated here and the prior cases, several concepts can be underlined, including the significance of individualized management and the significance of further research and improvements in spinal healthcare.

## Data Availability

The original contributions presented in the study are included in the article/Supplementary Material; further inquiries can be directed to the corresponding authors.
