# Supplementary material for: Case Report: Endoscopic cystectomy vs. lumbar interbody fusion for liquid- and gas-filled discal cysts: a case series and literature review
Source: Front Surg. 2025 Sep 11;12:1646605. doi: 10.3389/fsurg.2025.1646605 (PMC12460396; doi:10.3389/fsurg.2025.1646605)
Supplement: Supplementary file 1 [file Table1.docx]

Supplemental Table 1. Summary of the reported articles

| Article | Article type | Cases number | Level | symptom | Cysts type | Surgical strategy |
| --- | --- | --- | --- | --- | --- | --- |
| Msheik 2024 | Case report | 1 | L3/4 | severe leg pain | Liquid | Microdiscectomy and cystectomy |
| Ding 2024 | Retrospective Study | 7 | L3/4  L4/5  L5/S1 | Low back pain and radiating leg pain | Liquid | Endoscopic resection |
| Tan 2022 | Case report | 1 | L4/5 | Low back pain and radiating leg pain | Liquid | Endoscopic resection |
| Perillo 2022 | Case report | 1 | L3/4 | Low back pain and radiating leg pain | Gas | Microdiscectomy and cystectomy |
| Hu 2022 | Case report | 1 | L5/S1 | Low back pain and radiating leg pain | Gas | PELD |
| Zhu 2021 | Case report | 3 | L4/5 | Low back pain and radiating leg pain | Gas | TPED |
| Takamatsu 2021 | Case report | 1 | L4/5 | Low back pain and radiating leg pain | Liquid | Endoscopic resection |
| Suo 2020 | Case report | 2 | L4/5 | Low back pain and radiating leg pain | Liquid | Endoscopic resection |
| Chen 2020 | retrospective study | 9 | L3/4  L4/5  L5/S1 | Low back pain and radiating leg pain | Liquid | Endoscopic resection |
| Park 2019 | retrospective study | 27 | L2/3  L3/4  L4/5  L5/S1 | Low back pain and radiating leg pain | Liquid | Micro- cystectomy |
| Kim 2019 | Case report | 2 | L4/5  L5/S1 | Low back pain and radiating leg pain | Liquid | PELD |
| Aljuboori 2019 | Case report | 1 | L4/5 | Low back pain and radiating leg pain | Liquid | Micro- cystectomy |
| Sanjeevan 2018 | Case report | 1 | L4/5 | Low back pain and radiating leg pain | Liquid | Endoscopic resection |
| Mathon 2018 | Case report | 1 | L4/5 | Low back pain and radiating leg pain | Gas | TLIF |
| Cho 2016 | Case report | 1 | L5/S1 | Low back pain and radiating leg pain | Liquid | Micro- cystectomy |
| Bansil 2016 | Case report | 1 | L4/5 | Low back pain and radiating leg pain | Liquid | Micro- cystectomy |
| Jha 2015 | Case report | 1 | L4/5 | Low back pain and radiating leg pain | Liquid | Endoscopic resection |
| Certo 2014 | Case report | 1 | L3/4 | Low back pain and radiating leg pain | Liquid | Micro- cystectomy |
| Arslan 2014 | Case report | 1 | L3/4 | Low back pain and radiating leg pain | Gas | Micro- cystectomy |
| Wang 2013 | retrospective study | 9 | L2/3  L3/4  L4/5  L5/S1 | Low back pain and radiating leg pain | Gas | Micro- cystectomy |
| Yun 2012 | Case report | 2 | L4/5  L5/S1 | Low back pain and radiating leg pain | Gas | Micro- cystectomy |
| Lee 2012 | Case report | 2 | L4/5  L5/S1 | Low back pain and radiating leg pain | Gas | Micro- cystectomy |
| Kim 2012 | Case report | 1 | L4/5 | Low back pain and radiating leg pain | Liquid | Micro- cystectomy |
| Ha 2012 | retrospective study | 8 | L3/4  L4/5  L5/S1 | Low back pain and radiating leg pain | Liquid | Endoscopic resection |
| Kuh 2011 | retrospective study | 22 | L2/3  L3/4  L4/5  L5/S1 | Low back pain and radiating leg pain | Liquid | Micro- cystectomy or MIS-TLIF |
| Matsumoto 2010 | retrospective study | 7 | L3/4  L4/5  L5/S1 | Low back pain and radiating leg pain | Liquid | Endoscopic resection |
| Aydin 2010 | Case report | 5 | L1/2  L3/4  L5/S1 | Low back pain and radiating leg pain | Liquid | Micro- cystectomy |
| Kim 2009 | Case report | 2 | L4/5  L5/S1 | Low back pain and radiating leg pain | Liquid | Endoscopic resection |
| Kim 2009 | Case report | 1 | L5/S1 | Low back pain and radiating leg pain | Liquid | Endoscopic resection |
