# Supplementary material for: Case Report: Endoscopic cystectomy vs. lumbar interbody fusion for liquid- and gas-filled discal cysts: a case series and literature review
Source: Front Surg. 2025 Sep 11;12:1646605. doi: 10.3389/fsurg.2025.1646605 (PMC12460396; doi:10.3389/fsurg.2025.1646605)
Supplement: Supplementary file 2 [file Table2.docx]

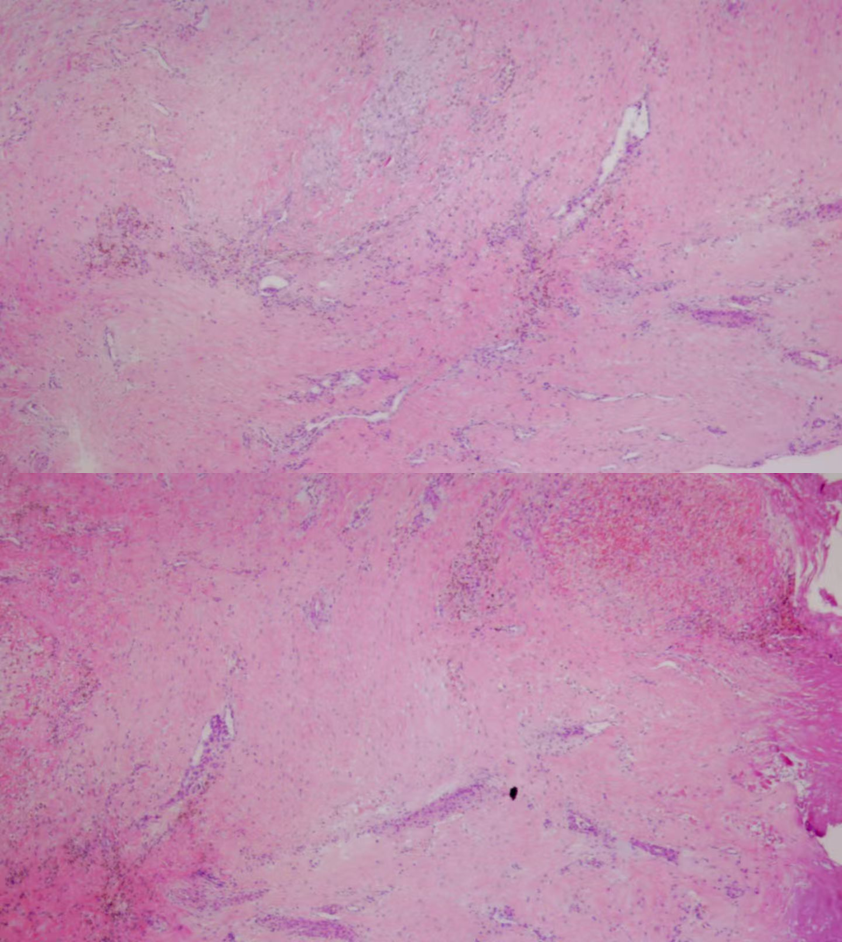


Supplemental Figure 1: The tissue of the cyst is fibrous and vascular, with fibrous tissue hyperplasia and hygroma, and focal old hemorrhage
